# DNA Polymerases at the Eukaryotic Replication Fork Thirty Years after: Connection to Cancer

**DOI:** 10.3390/cancers12123489

**Published:** 2020-11-24

**Authors:** Youri I. Pavlov, Anna S. Zhuk, Elena I. Stepchenkova

**Affiliations:** 1 Eppley Institute for Research in Cancer and Allied Diseases and Buffett Cancer Center, University of Nebraska Medical Center, Omaha, NE 68198, USA; 2 Department of Genetics and Biotechnology, Saint-Petersburg State University, 199034 Saint Petersburg, Russia; stepchenkova@gmail.com; 3 International Laboratory of Computer Technologies, ITMO University, 197101 Saint Petersburg, Russia; ania.zhuk@gmail.com; 4 Laboratory of Mutagenesis and Genetic Toxicology, Vavilov Institute of General Genetics, Saint-Petersburg Branch, Russian Academy of Sciences, 199034 Saint Petersburg, Russia

**Keywords:** DNA polymerases, proofreading exonucleases, replication fidelity, mutation rates, cancer predisposition

## Abstract

**Simple Summary:**

The etiology of cancer is linked to the occurrence of mutations during the reduplication of genetic material. Mutations leading to low replication fidelity are the culprits of many hereditary and sporadic cancers. The archetype of the current model of replication fork was proposed 30 years ago. In the sequel to our 2010 review with the words “years after” in the title inspired by A. Dumas’s novels, we go over new developments in the DNA replication field and analyze how they help elucidate the effects of the genetic variants of DNA polymerases on cancer.

**Abstract:**

Recent studies on tumor genomes revealed that mutations in genes of replicative DNA polymerases cause a predisposition for cancer by increasing genome instability. The past 10 years have uncovered exciting details about the structure and function of replicative DNA polymerases and the replication fork organization. The principal idea of participation of different polymerases in specific transactions at the fork proposed by Morrison and coauthors 30 years ago and later named “division of labor,” remains standing, with an amendment of the broader role of polymerase δ in the replication of both the lagging and leading DNA strands. However, cancer-associated mutations predominantly affect the catalytic subunit of polymerase ε that participates in leading strand DNA synthesis. We analyze how new findings in the DNA replication field help elucidate the polymerase variants’ effects on cancer.

## 1. Prologue: Mutations in DNA Pol Genes and Cancer

**Figure 1:**
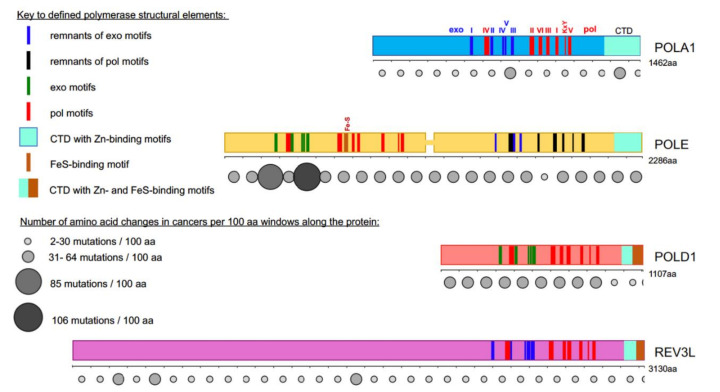
Most cancer-associated mutations affect the catalytically active half of POLE. Colored bars represent the main subunits of DNA pols, a catalytic subunit of pol α, POLA1, in light blue; of pol ε, POLE, in yellow; of pol δ, POLD1, in red; and pol ζ, REV3L, in purple. Note that POLE is a tandem of active pol (*N*-terminal half) and inactive pol (C-terminal half) [5,6]. Evolutionarily conserved motifs characteristic for all exonuclease (exo) domains, are labeled I-V in green and pol domains are labeled I-VI and KxY in red [5,6,7,8,9,10,11,12]. The order of the motifs along all four proteins is the same, but they occupy different parts of the whole protein. For example, REV3L has a very long *N*-terminal part not related to pols. In POL1, REV3L, and the C-terminal half of POLE, the exonuclease motifs are inactivated during evolution; they are shown in blue. Inactivated pol motifs in the C-terminal half of POLE are shown in black. The key for these and other elements of the pol primary structure is in the left upper quarter of the figure. Rows of circles of different sizes and shades of grey below the catalytic pol subunits represent the number of missense mutations found in tumors along the protein regions in 100 amino acid increments. Variants were collected from the cBioPortal database from a curated non-overlapping collection of tumor genomes (https://www.cbioportal.org/ (cbioportal.org)). A guide explaining the relation between size and intensity of grey to the number of mutations found in the database in the 100 amino acids interval is on the left lower quarter of the figure.

Research in the past decade has revealed the lofty role of alterations in replicative DNA polymerases (pols) in sporadic and hereditary cancer [1,2]. The predisposition to tumorigenesis is attributed to the low fidelity of DNA replication by inaccurate pol versions [3,4]. Among the replicative B-family enzymes, pol ε stands out. The alterations in the proofreading exonuclease domain caused by mutations in the *POLE* gene (see Table 1 for the nomenclature of DNA polymerase subunits in humans and in yeast and mouse models) are proven to be causative factors in the etiology of the malignant transformation (Figure 1), with predominant, but not exclusive prevalence, in colon and endometrial cancers. The review analyzes how the modern understanding of the replication fork based on the synthesis of information gained in model systems and genomics of tumors may explain the peculiarities of the connection of pols and cancer in humans.

## 2. Loss of Replication Fidelity Control Elevates Mutation Rates: Classic Rules

Three steps, base selection, exonucleolytic proofreading, and DNA mismatch repair (MMR), ensure the high fidelity of DNA replication [13,14]. As determined first in the microorganism’s models, if one of the three steps is inefficient, mutation rates elevate 10–100-fold. In yeast, low base selectivity caused by amino acid changes in pol region II (mutation is in *POL1* [15], *POL3* [16,17], *POL2* [18], and *REV3* [19], Figure 1) leads to increases in spontaneous mutation rates. These variants are called “mutators”. Two pols, pol δ and pol ε, possess a functional exonuclease (exo) domain and correct replication errors. The substitutions of amino acids responsible for the exo activity (exo dead variants, exo^-^) (exo region I, Figure 1) lead to less than a ten-fold mutator effect when happening in Pol2 [20] and up to a 100-fold mutator effect when happening in Pol3 [21]. It is interesting to note that strains with equally exo dead pol δ due to changes in exo motif III are only 20-fold mutators [22], suggesting that the absence of exo activity by itself does not accurately predict the mutator effect. In the case of a mutant with changes in catalytic residues in motif I in Pol3, checkpoint involvement in the very high mutation rate has been proposed [23].

Because three fidelity steps occur in a series, a combination of defects in any of the two consecutive steps results in multiplicative, more than a 1000-fold increase of mutation rates, up to levels that are incompatible with the life of haploids: exo^−^ pol ε or pol δ with MMR defect (MMR^-^) [21]; exo^-^ pol ε with exo^-^ pol δ [24,25]; relaxed base selectivity of pol α, or pol ε, or pol δ with MMR^-^ [15,26,27]; and low base selectivity of pols ε or δ with their proofreading defects [25,28].

## 3. The Cornerstone Model of the Replication Fork

**Figure 2:**
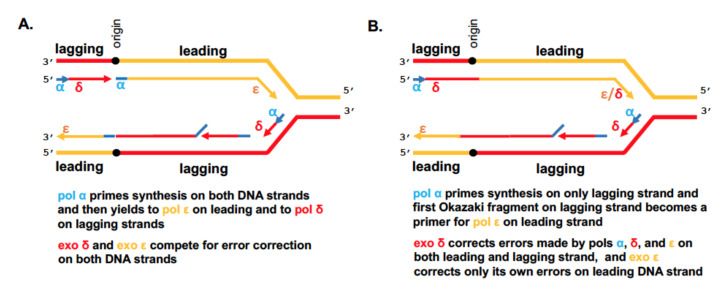
The replication fork seen in 1990 looks almost right in 2020. (**A**) Schematic representation of the model proposed by Morrison et al. in 1990 [29]. The bidirectional replication starts at the origin, and part of the fork moving to the left is not shown. Primase/Pol α synthesizes short RNA/DNA primers extended by pol ε on the leading strand and by pol δ on the lagging strand. Most of these primers are excised from the newly synthesized DNA [31]. Proofreading exonucleases associated with pol δ and ε have access to 3′-DNA ends on both strands and thus compete to proofread replication errors [24]. The mismatch repair step is not shown for simplicity. (**B**) The current vision of replication fork. Pol ε does not participate in any transactions on the lagging DNA strand. Pol δ and pol α contribute to the replication of both strands. It is estimated that only 1.5% of DNA synthesized by primase-pol α is retained in newly synthesized DNA in humans [32]. Moreover, 80% of the leading strand is synthesized by pol ε. Pol δ synthesized DNA is at least 18% of the leading [33], and more than 90% of lagging DNA strands [34,35].

In 1990, a *Cell* paper described the discovery of the third replicative DNA polymerase, pol ε in yeast [29], and a paper in the *Proceeding of the National Academy of U.S.A.* characterized pol ε purified from HeLa cells [30]. While trying to find the answer to why the eukaryotic cell needs three pols to replicate its DNA, Morrison and coauthors [29] ingeniously proposed that “each of the three polymerases is specialized for one of the different modes of synthesis required as a replication fork moves from a specific origin”. Τhe presence of the proofreading exo domain (thus a potential to correct replication errors as pol δ can do) and the high processivity of pol ε led Morrison and coauthors to a simple model where pol ε synthesizes leading and pol δ synthesizes the lagging DNA strands (Figure 2A). Genetic experiments suggested that exos of both pols δ and ε can compete for the same pool of replication errors, but it was not clear if they can freely correct errors made by another pol [24]. The role of pol α was, together with primase, to synthesize short RNA-DNA hybrids as primers in leading and lagging DNA synthesis (Figure 2A).

The first evidence in favor of the model came from genetic experiments in yeast when it was demonstrated that the proofreading exonucleases of pol δ and pol ε correct replication errors on different DNA strands [36,37]. After almost a 17-year lag in searches for the truth (examples of different models: [38,39,40,41]), this model became generally accepted, driven by the fact that eukaryotic helicase CMG complexed with pol ε travels along the leading strand, and was backed by genetic and biochemical data [42,43]. Recently, it has been updated by acknowledging the role of pol δ in leading strand synthesis [35] (Figure 2B), as we proposed in our previous review on the subject a decade ago [44]. It is conceivable that short RNA-DNA fragments synthesized by primase/pol α and pol δ during the start of lagging strand synthesis serve as a start of leading strands, thus evading the need for a separate mechanism for pol α/pol ε switch (Figure 2B) [45]. Pol δ might participate in DNA synthesis at the replication termination zones [46]. Also, Pol δ operates on the leading DNA strand after replication restart when DNA is damaged [47]. Finally, pol δ proofreads errors not only on the lagging but also on the leading DNA strands, while pol ε is strictly assigned to the leading strand (Figure 2B) [25,44,48] and it is unimaginable that after successful proofreading the pol does not continue DNA synthesis to some extent on the same strand.

We can conclude that the best description of pols’ arrangement at the fork is that three pols synthesize most genomic DNA, but pol ε is excluded from the lagging DNA strand transactions, being helicase-associated leading strand DNA polymerase [35]. Therefore, yeast strains without the catalytic half of Pol2 but with the C-terminal part bound to CMG are viable but have a severe growth defect [49,50,51,52], suggesting that pol δ can completely substitute for the missing helicase-associated pol, albeit with reduced effectiveness. Indeed, pol δ can synthesize both strands in more natural circumstances, during viral replication [39] or during break-induced replication [53]. Moreover, mutations in the cyclin-dependent kinase gene, *CDC28,* restore near-normal growth characteristics of strains without the catalytic half of Pol2, implying that cell cycle control machinery can facilely accommodate for its absence [54]. The connection between CDKs and pol ε has also been demonstrated for breast cancer cell lines [55].

## 4. Progress on the Structure-Function of B-Family DNA Polymerases and Organization of the Replication Fork

**Figure 3:**
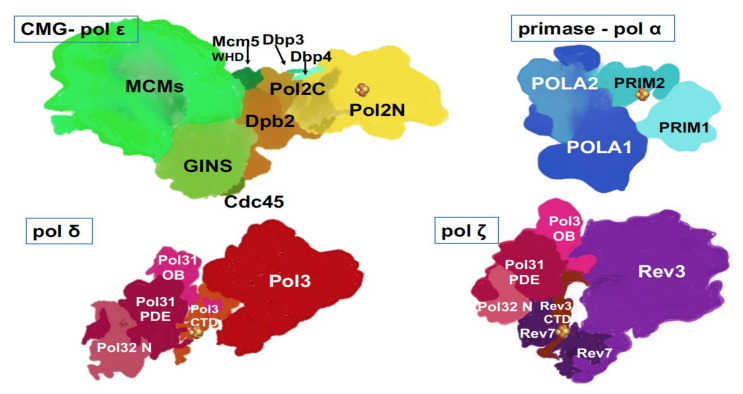
Multi-subunit replicative DNA pols. Artistic representations were made based on crystal and cryo-EM structures and models of human primase-pol α [58], yeast pol ε [65,67], yeast pol δ [60], yeast pol ζ [66]. Two latter structures were determined with a truncated third subunit (Table 1), Pol 32, without the C-terminal part, and thus, this part is missing from our drawings. Fe-S cluster (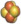) is present in each of the four pol complexes.

The past ten years brought groundbreaking discoveries about B-family DNA pols. Currently, with the help of X-ray crystallography and the improvement of cryo-EM resolution, we understand the atomic details of the structures of catalytic cores and whole complexes (Table 1) of yeast and human (Table 1) primase-pol α [56,57,58], yeast and human pol δ [59,60,61], yeast pol ε alone or in complex with CMG [62,63,64,65], yeast pol ζ [66] (Figure 3).

As we can see from the list, two human pols’ structures can only be modeled based on solved yeast counterparts, thus making the solution of structures of human pols a high priority. The structures of yeast and human pols appear to be similar in general features but differ in nuances. For example, human pol δ has an additional small subunit, p12 (Table 1) hypothesized to regulate pol δ activity during normal replication versus conditions of DNA damage or replicative stress [61,68]. The catalytic subunit of human pol ζ has extended the *N*-terminal part of unknown significance (Table 1, Figure 1 and Figure 3). Structural and functional studies helped understand transactions in the active site of polymerases and within the pol complexes. Examples of success are the basis of RNA primer synthesis by primase and transfer of RNA primer 3′-end into pol α active site to start DNA synthesis; the reasons for the high fidelity of pols δ and ε; and the ability of pol ζ to extend mismatches or unpaired DNA ends found opposite lesions.

One exciting finding is that all DNA pols coordinate Fe-S clusters, known regulatory/structural elements of various proteins [69], alluding to the connection of iron metabolism in mitochondria to replication and novel opportunities for regulation of pol reactions (Figure 1 and Figure 3) [70,71]. The cluster can accept or donate electrons and might be involved in sensing the redox potential of cells and DNA damage [72,73]. The first finding was the detection of the Fe-S cluster in the second subunit of archaeal and yeast primase [74], which was proven to play a seminal role in the primer synthesis by human enzyme [58,75,76,77]. Then, Fe-S clusters were found and verified in C-terminal regions of yeast and human pols δ and ζ (Figure 1 and Figure 3**)** and were shown to be necessary for pol function [70,78,79]. The Fe-S cluster in the catalytic subunit of pol ε was found in an unusual location: in the *N*-terminal half in the vicinity of pol II motif (Figure 1), structurally characterized, and shown to be necessary for pol but not exo activity in functional assays [80,81]. A recent study revealed the unique sensitivity of pol ε to suppression of Fe-S biosynthesis in basal-like breast cancer cell lines [55].

Another sensational discovery was the sharing of subunits between pols δ and ζ [78], Table 1, Figure 3. For quite an extended period, pol ζ was referred to as a two subunit enzyme consisting of catalytic Rev3 and accessory subunit Rev7 [82], later found to be one REV3 to two REV7 subunit complex [66,83]. The two-subunit complex possessed quite low and variable activity [84,85,86]. The pol δ’s two accessory subunits appeared to be two additional subunits of pol ζ necessary for the full activity [78,87,88,89]. It appeared that the inconsistent activity of former “two-subunit” preps resulted from uncontrolled traces of a genuine four subunit enzyme [88]. The role of such subunit sharing between the main replicative pols and pol ζ is under debate. In the original paper describing the discovery, an elegant mechanism of switches of pol’s catalytic subunits on the already present core of PCNA/POLD2/POLD3 was proposed [78], and the role of Fe-S clusters in CTDs of both pols recognized [90,91], but possible details of the process have never been elaborated. The argument against the switch mechanism is the stability of multi-subunit complexes of pols δ and ζ [87,88]. Pols’ architecture with shared subunits might reflect evolutionary relationships and structural requirements [66].

**Figure 4:**
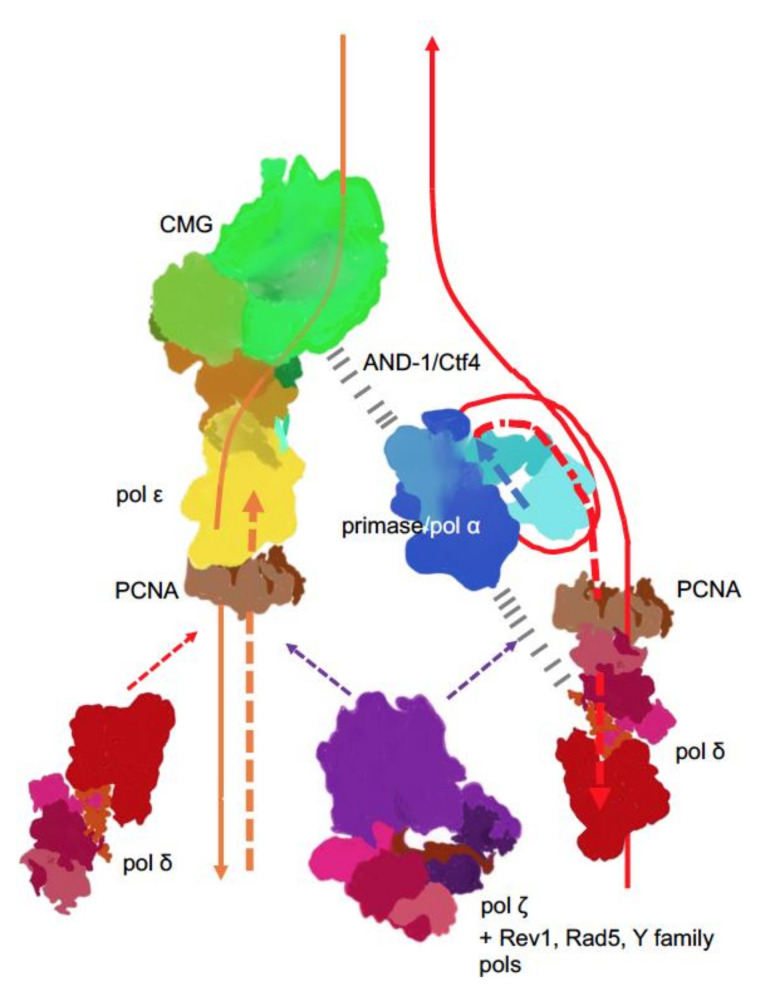
Main players at the eukaryotic replication fork. The CMG complex unwinds DNA, primase-Pol α synthesizes short RNA-DNA primers that are extended by pol δ. On the lagging strand, pol δ synthesis is halted when the pol reaches the previous Okazaki fragment. On the leading strand, pol ε takes over and contributes to around 80% of the bulk strand synthesis. Pol δ occasionally proofreads errors made by pol ε [25] and likely continues synthesis on the leading strand thereafter. The coordination of the whole process is likely achieved by interactions of primase-pol α with CMG via Ctf4 (yeast) or AND-1 (humans) [93,94,95,96] and some not precisely mapped pol α interactions with pol δ (dashed black lines) [97,98,99]. Replication stress caused by unusual DNA structures [100], DNA damage or defects in replisome [101,102,103,104] lead to recruitment and patches of synthesis of the fourth member of the B-family, pol ζ [105], along with translesion pols and accessory factors to mitigate replication problems, depending on the nature of replication problems.

New findings lead to a better understanding of replication fork in eukaryotes (Figure 4). The CMG complex bound to the C-terminal part of pol ε travels along on the leading strand. This tight association explains the participation of pol ε in the leading strand synthesis and exclusion of this pol from synthesis and proofreading on the lagging strand. In yeast pol, the accessory subunits of Pol2, Dpb3/Dpb4, may serve as “staples” rigidly connecting the C-terminal part with the active *N*-terminal part [65]. However, if this rigidity were stable, any transactions by other DNA pols on the leading strand (for example, when the switch to translesion pol is necessary for DNA damage bypass) would have been blocked by the *N*-terminal half of Pol2 stuck with the primer terminus, but this is not the case. Pol δ proofreads errors made by pol ε [25,44] and pol ζ, with other translesion DNA synthesis pols, operate on the leading strand to the same extent as on the lagging strand [44,92]. Therefore, there should be a mechanism of how the active part of the catalytic subunit of pol ε abandons the 3’-end of the nascent leading strand and yields to other pols.

The lagging DNA strand is synthesized in relatively short Okazaki fragments whose size coincides with the nucleosomal repeat (165 bp), as measured under conditions of constrained ligation [106,107]. The evidence from the distribution of inaccurate pol α-dependent mutations in yeast seems to support this estimate [108]. The need for the ligation of short DNA fragments on the lagging strand led to a straightforward assumption that nicks and ssDNA regions are more prevalent in this strand. Such a property of the lagging strand would explain the more efficient operation of MMR on the lagging strand [109], or preferential damage of the lagging strand by DNA editing cytosine deaminases of the APOBEC family that act on ssDNA, shown in model systems [110,111] and tumors [112,113]. However, recent findings suggest that the leading DNA strand is discontinuous as well, and nicks in the yeast’s leading strand are even more frequent than in the lagging strand [107]. The effect is attributed to ribonucleotide excision, as seen in bacteria [114,115] and the preferential incorporation and repair of ribonucleotides in the leading DNA strand in yeast.

## 5. DNA Polymerase Genes Mutations in Cancer

Defects in MMR for a long time were the only factors in hereditary non-polyposis, endometrial, and other cancers in Lynch syndrome, connecting replication fidelity to cancer [116,117,118,119]. The topic of MMR role in cancer is extensively discussed and reviewed [116,120,121,122] and is not touched here. Recent studies of cancer genomes discovered mutations in genes of the DNA pols of the B family in many sporadic and hereditary cancers [1,2] (Figure 1). Mutations affect all four DNA pols (Figure 1). For most, their functional significance in malignant transformation is unknown. The exception is mutations that affect the POLE and POLD1 proofreading domains connected to ultra-mutated sporadic and hereditary tumors (Figure 1) [2,123]. The studies of such mutations revealed paradoxical facts not fully understandable in the frame of our current view on pols’ roles and properties of at the fork.

The first question is: does the defect of proofreading exonuclease predict a high mutator effect and a prerequisite for malignant transformation? There is a general correlation between the mutator effect in the model system and the particular allele’s frequency in cancer [2]. When mutations in *POLE* were first discovered, their effect was hastily attributed to pol ε’s inability to correct errors and thus lower replication fidelity [1,124]. The idea was consistent with the knowledge in model systems because the complete defect of pol ε exo activity when two catalytic aspartates are changed to alanine increases the mutation rate in yeast and causes a mutator effect and cancer predisposition in mice (Table 2, row 1). However, half of the prominent mutations in human cancers do not entirely abolish exo activity but possess a superior effect on mutation rates and cancer incidence than “golden standard” mutation leading to change D292A;E294A. Change P286R is the most abundant in sporadic ultra-mutated tumors (Table 2, row 3). The mutator effect of its yeast homolog is incredibly high; mice homozygous for the analogous change do not survive while heterozygous mice rapidly develop cancers, although their types do not recapitulate human cancer types (Table 2, row 3). The pol ε with the change, however, has residual exo activity. Variants V411L and L424V are found in hypermutated tumors and predispose for sporadic and hereditary cancers, but corresponding enzymes have a substantial exo activity (Table 2, rows 8,9). P286R is quite intensively modeled in yeast [125]. The mutator effect of the change stunningly exceeds the mutator effect of classic exo deficiency caused by allele *pol2–4* by almost two orders of magnitude [126]. However, the purified yeast pol ε with the corresponding P301R change is surprisingly more accurate in vitro than exonuclease defective D292A;E294A enzyme (though, of course, less accurate than exo^+^ pol ε), and produced a spectrum of mutations that was not drastically different [125]. Therefore, the effect could only be seen in vivo. Several hypotheses have been proposed to explain the paradox. The first idea relies on the “division of labor” between pols ε and δ. We can hypothesize that the current vision that pol ε synthesizes most of the leading strand is wrong, and pol ε works mostly near replication origins and later yields to pol δ [44]. If pol ε P301R synthesizes much more DNA, its mutator effect will exceed the exo defective pol’s mutator effect. The hypothesis predicts that commonly observed mutation bias, attributed to different properties of pols ε and δ [127] should disappear with the increase of the distance from the origin. However, mutation bias at different locations along the replicon was similar in *pol2-P2301R* and exo^-^ *pol2–4* strains, and the idea was dismissed [128]. The second hypothesis is that pol ε P301R somehow prevents extrinsic proofreading by pol δ [25]. For example, it is possible if the “rigidity” [65] of pol ε P301R is much greater than other pol variants. This hypothesis is ruled out by the multiplicative increase of mutation rates in *pol2-P301R* strains combined with a proofreading defect of pol δ, allele *pol3–5DV* (Table 2, last row) [128]. Similarly, *pol2-P301R* is multiplicative with MMR defects, implying that MMR is operational to the full extent on DNA synthesized by pol ε P301R. The third, current working hypothesis, is based on the solved structure of the mutant variant and biochemical finding of the elevated pol activity of P301R pol. The change alters the structure of Pol2 in such a way that the access of 3’-end of the nascent DNA chain to the exo site is blocked [129]. As a result, the enzyme does not waste time partitioning between pol and exo sites [130,131] and robustly extends mismatches during the synthesis [125]. To reconcile a huge mutator effect with a high in vitro fidelity, we should postulate that the enzyme’s unique properties manifest only during replication in live cells. It is currently unclear how similar logic could be applied to other cancer-associated mutations in the exo domain, whose mutator effect exceeds the mutator effect of *pol2–4* (Table 2).

Other explanations of the very high mutator effects of mimics of some cancer-associated mutations in yeast (e.g., P301R, S474F, and the high mutator effects of other variants, Table 2) could be considered, but, so far, they have not been tested. The simplest one is that pol ε D290A;E292A is an outlier and does not represent a typical pol ε proofreading failure. For example, if, in addition to an exo defect, the enzyme possesses some other defects masking its involvement in replication. The most powerful mutator effect (among cancer-associated mutations modeled in yeast) in strains with pol ε P301R is not understandable because of some proofreading activity compared to, for example, pol ε S474F (Table 2). If we admit almost the same fidelity of pol ε P301R and pol ε D290A;E292, then the difference in mutation rates of the strains with these pols means that, at a given site of the genome, the probability of a fixating replication error is around hundred times more in strains with pol ε P301R than in strains with the pol ε D290A;E292 variant. This is hard to explain in the frame of a strict “division of labor” postulate. Perhaps the arrangement of pols at the fork in different cells/genome regions fluctuates, and P301R change leads to a shift for preferential action of pol ε. The superior activity of pol ε P301R is consistent with the idea. The selection of yeast variants that relied on the robust growth of pol exo-variants gave a high proportion of mutants that correspond to mutants found in human cancers [132].

Another puzzle is why *POLE* mutations in the exo-coding part overwhelmingly outnumber *POLD1* mutations in cancers if these pols both replicate the whole genome with comparable fidelity [134,144], but on different strands. If pol δ proofreads on both strands, we expect more potent effects of exo defects in pol δ, precisely what was observed in yeast for classic exo^-^ mutations, causing the change of the two catalytic residues in Exo I motifs of pols ε and δ (Table 2, compare rows 1 and 10). It could be argued that mutations affecting amino acids in pol δ analogous to exo^-^ variants in pol ε could never be found in cancers because they have too strong mutator effects incompatible with the cells’ functions. High mutation rates lead to catastrophic accumulation of mutations and cell death [24,28,128]. Consistently, the hypermutator alleles encoding for the analog of P286R change in homozygous state are inviable in mice, while causing cancer when heterozygous [122,135] (Table 2, two last columns of row 3). In the critique of this explanation, we can note that different mutations causing defects of proofreading exo cause different increases of mutation rates (Table 2), and thus it is not clear why tumors do not accumulate “mild” or “leaky” alleles, moderately affecting the exo function of pol δ. Another explanation is that cells with severe proofreading defects by pol δ rapidly turn on mechanisms suppressing mutator effects [136,137]. Some support comes from the mouse model, where, contrary to the expectations, mutation rates in cells from pol δ exo^-^ mice are lower than from pol ε exo^-^ [138]. However, the direct comparison is impossible because these mice have different mutations affecting the exo domain, causing changes in Exo I in POLE but Exo II in POLd1 mice. It is still a puzzle why pol δ exo^-^ mice accumulate completely different tumors comparing to pol ε exo^-^ mice (Table 2, last column). Also, we know from yeast, mouse and human cancers that different exo defects might have very different consequences (Table 2). It is important to note that the spectrum of accumulated tumors in mice with pol variants differs from human cancers supposedly caused by similar variants. Thus, the mice model is good for studying the correlation between elevated mutation rates and tumorigenesis, but does not recapitulate tissue-specific carcinogenesis in humans.

Two cancer-associated changes in the exo domain of POLE deserve special attention, V411L, and L424V (Table 2, rows 8 and 9). These changes do not abolish exo activity. The change V411L is the second most frequent mutation in sporadic ultra-mutated cancers, and was even found in hereditary cancers (rarely), but it is not a mutator when modeled in yeast [2]. A straightforward explanation is that the structures and properties of the corresponding yeast and human enzymes with the change are different. The controversy might be resolved when the structure of the human POLE and its V411L variant will be available. More complicated scenarios predict that the downstream pathways of mutagenesis affected by the change are different in yeast and humans. L424V is a modest mutator in yeast, frequently found in cancers with a striking bias to hereditary cancers. The innovative idea is that the L424V allele might be a hotspot for spontaneous germ-line mutagenesis [2] because of the site’s location near the base of a putative hairpin, known as a hotspot of Rev1-pol ζ dependent DRIM mutagenesis [100].

## 6. Conclusions: A Projection into the Future

Tumor genomes databases list thousands of mutations in pol genes. It is conceivable that the vast majority are passengers. We predict that, along with further progress and accuracy in the characterization of tumor genomes and functional characterization of recurrent mutations, new regions of DNA pols catalytic and other subunits whose alterations predispose to cancer will be found. One example of the significance of *POLD1* polymerase domain alterations in colorectal cancer is a mutation leading to R689W change in pol III motif [147,148]. The knockout of the gene for an accessory subunit of pol ε, *Pole4* in mice causes genome instability and elevated tumorigenesis if Tp53 is also knocked out [149]. *Rev3l^−/−^* mice are inviable, but MEF lines with the addition of *Tp53^−/−^* exhibit striking genome instability [150]. It is likely that cancer-associated mutations will be found in the genes encoding pol ζ, which is responsible for mutagenesis by virtually all DNA-damaging agents. We will learn more about the functionality of mutations in genes for pols and other components of cells, assuring genome integrity, and a more precise and detailed list of cancer susceptibility pol alleles. Modeling the mutations in mice will provide more sophisticated information on their biological consequences in comparison to the yeast model. Structural studies of human pols by crystallography and EM will help to define all critical regions of DNA pols responsible for the fidelity of replication and interaction with partners.

## Figures and Tables

**Table 1:** Nomenclature of subunits of yeast and human B-family DNA polymerases *.

Polymerase	Subunit **	Yeast, *S. cerevisiae*			Human, *H. sapiens*		
		Gene	Protein, (Size, kDa)	Also Known As:	Gene	Protein, (Size, kDa)	Also Known As:
primase -pol α	Small (catalytic primase)	*PRI1*	Pri1 (48)	YIR008C	*PRIM1*	PRIM1 (50)	p48, p49
	Large of primase	*PRI2*	Pri2 (62)	YKL045W	*PRIM2*	PRIM2 (59)	*PRIM2A*, p58
	Catalytic	*POL1*	Pol1 (167)	*CDC17, CRT5, HPR3,* YNL102W	*POLA1*	POLA1 (166)	*POLA, NSX*, p180
	B-subunit	*POL12*	Pol12 (79)	YBL035C	*POLA2*	POLA2 (66)	*FLJ21662,* p70
pol ε	Catalytic	*POL2*	Pol2 (256)	*DUN2,* YNL262W	*POLE*	POLE (262)	*FILS,* *POLE1*, *IMADEI*, *CRCS12*, p261
	B-subunit	*DPB2*	Dpb2 (78)	YPR175W	*POLE2*	POLE2 (60)	*DPE2*, p59
	Third	*DPB3*	Dpb3 (23)	YBR278W	*POLE3*	POLE3 (17)	*CHRAC2*, *YBL1*, p17
	Fourth	*DPB4*	Dpb4 (22)	YDR121W	*POLE4*	POLE4 (12)	*YHHQ1*, p12
pol δ	Catalytic	*POL3*	Pol3 (125)	*CDC2, HPR2, TEX1*, YDL102W	*POLD1*	POLD1 (124)	*CDC2, MDPL, CRCS10*, p125
	B-subunit	*POL31*	Pol31 (55)	*HYS2, HUS2, SDP5*, YJR006W	*POLD2*	POLD2 (51)	p50
	Third	*POL32*	Pol32 (40)	*REV5*, YJR043C	*POLD3*	POLD3 (51)	*PPP1R128, KIAA0039*, p66
	Fourth	*-*	*-*		*POLD4*	POLD4 (12)	*POLDS*, p12
pol ζ	Catalytic	*REV3*	Rev3 (173)	*PSO1*, YPL167C	*REV3L*	REV3L (353)	*REV3*, *HREV3, POLZ* p353
	Second	*REV7*	Rev7 (29)	YIL139C	*hREV7*	HREV7 (24)	*MAD2L2*, p30, *REV7*, *FANCV*, *MAD28,* *POLZ2*,
	B-subunit	*POL31*	Pol31 (55)	*HYS2, HUS2, SDP5*	*POLD3*	POLD3 (50)	p50
	Fourth	*POL32*	Pol32 (40)	*REV5*	*POLD4*	POLD4 (12)	*PPP1R128*, p66

*—mouse gene symbols are the same as humans but written using different capitalization: example for a gene is *Pole*, for a protein—POLe. **—the information on catalytic subunits is highlighted by bold font.

**Table 2:** Examples of mutator and carcinogenic potential of *POLE* and *POLD1* exo domain mutations in humans and in model systems.

Pol Amino Acid Change; (Exo Activity) ^§^	Pol Region, Sequence, Conservation. Changed Amino Acids Are Underlined. Amino Acids Different from Human Protein Are Highlighted Grey.	Recurrently Found in Cancers *; Predominant Types of Cancer [124,139] and cBioPortal	Mutation Burden in Genomes of Tumors with the Change **	Sporadic (s) or Hereditary (h)	Yeast Variants:			Mice Variants:
					Allele Name; Amino Acid Change; (Exo Activity)	Mutator Effect Relative to Wild-Type; (Method of Determination)	Allele Variant (Exo Activity)	Mutator Effects Relative to Wild-Type; (Method of Determination); Cancer Incidence; Predominant Types of Cancer
POLE								
D275A; E277A; (-) [122,140]	Exo I Hs PVVLAFDIETTKLP Mm PVVLAFDIETTKLP Sc PVVMAFDIETTKPP	not found, classic model exo^−^ variant	n/a	n/a	*pol2–4;* D290A;E292A; (-) [125]	5; (Can^r^) [20] 2.9; (Can^r^) [126] 4; (SNVs /genome) [141] ~11; (Lyp^-^) [141]	*Pole^D272A;^^E274A/D272,E274A^*(-) ***	10; (derivatives of BigBlue^TM^ mice); [138] >70; (ouabain resistance) or 170; thioguanine resistance in MEFs) [138] ;medium; intestine adenocarcinoma, nodal lymphoma [138]
D275V; (-) ***D275A; (-)	Exo I Hs PVVLAFDIETTKLP Mm PVVLAFDIETTKLP Sc PVVMAFDIETTKPP	+; endometrial, breast, glioblastoma, colorectal, lung	med	s	*pol2-D290V;* (-)	2.3; (Can^r^) [133] ~9; (Lyp^-^) [141]		
P286R; (−/+) [122]	Exo I Hs FDIETTKLPLKFPD Mm FDIETTKLPLKFPD Sc FDIETTKPPLKFPD	+++; colon, endometrial, ovarian, high grade glyoma pancreatic, breast, prostate, bladder and other cancers	ultra-high	s	P301R; (-/+) [125]	150; (Can^r^) [126]~200; (Lyp^-^) [141] 63; (SNVs/ genome) [141]	*Pole^P286R/+^* (P286R; nd	~40; (NGS ^#^) [135] >100; (NGS) [122]; high; thymic lymphomas, lung adenocarcinomas, angiosarcoma [135]; thymic lymphomas, splenic lymphomas [122]
P286H; (−/+) [122,142] P286L	Same region, but different amino acid change	+; colon, glioblastoma, stomach	ultra-high	s	P301H; nd	13; (Can^r^) [133]		
S459F; (-) [122,142]	ExoIII Hs TYSVSDAVATYY Mm TYSVSDAVATYY Sc EYSVSDAVATYY	++; colon, endometrial, glioblastoma, duodenal	high	s	S474F; nd	30; (Can^r^) [133]	*Pole^S459F/S459F^* (S459F); nd	>220; (NGS); high; thymic/splenic lymphomas [122]
F367S; (+/−) [122,142]	Exo II Hs MVTYNGDFFDWPF Mm MVTYNGDFFDWPF Sc ISTFNGDFFDWPF	+; endometrial, colon	ultra-high	s	F382S; nd	17; (Can^r^) [133]		
P436R; nd	Exo V Hs AKLGYDPVELDP Mm AKLGYDPVELDP Sc AKLGYNPIELDP	+; endometrial, colorectal	ultra-high	s	P451R; nd	5.2; (Can^r^) [133]		
V411L; (+/-) [142]	Hs CLRWVKRDSYLPV Mm CLRWVKRDSYLPV Sc CFRWVKRDSYLPQ	+++; +; endometrial, colorectal, glioblastoma, kidney cancer, ovarian medulloblastoma, urinary tract, cervix, stomach	ultra-high	s h	V426L; nd	1.2; (Can^r^) [133] ~5; (Lyp^-^) [141] 1.8; (SNVs/ genome) [141]		
L424V; (+/-) [122,142]	Exo IV Hs LPVGSHNLKAAAK Mm LPVGSHNLKAAAK Sc LPQGSQGLKAVTQ	+; +++; colorectal, endometrial, lung, breast, glyoblastoma, duodenal [122]	med	s h	L439V; nd	5.2; (Can^r^) [133] ~33; (Lyp^-^) [141] 7; (SNVs/ genome) [141]		
POLD1								
D316A; E318A; (-)	Exo I Hs LRVLSFDIECAGRK Mm LRVLSFDIECAGRK Sc LRIMSFDIECAGRI	Double change not found, but +; D316N or D316G; endometrial	high	s	*pol3–01*; D321A;E323A; (+) [22]	130; (FOA^r^) [21] 110; (Can^r^) [22]		
D402A; (-) [143]	ExoII Hs TGYNIQNFDLPYLI Mm TGYNIQNFDLPYLI Sc TGYNTTNFDIPYLL	not found in cBio, but other changes of the nearby amino acids in motif +; breast adeno carcinoma, melanoma	Q399H med P404Shigh	s	*pol3–4DA;* D407A; (-) [144] *pol3–4DV;* D407V;nd	55; (Can^r^) [22] 76; (Can^r^) [22]	*Pold1^D400A/D400A^* (-)[145]	3; (derivatives of BigBlue^TM^ mice) [138] ;>10; (ouabain resistance in MEFs) [145] 6; (fibrosarcoma cell line) [145] >50; (ouabain or thioguanine resistance in MEFs) [138] ;high; thymic lymphomas, tail skin carcinoma, lung adenocarcinoma
D515A; (-) [146]	Exo III Hs AVYCLKDAYLPLRL Mm AVYCLKDAFLPLRL Sc AVYCLKDAYLPLRL	+; currently not found, but D515N variant was detected in melanoma	med	s	*pol3–5DV*; D520V; (-) [22]	20; (Can^r^) [22]		

^§^ Exo activity: (-)—none; (−/+)- residual; (+/−)—detectable, around 50% of wild-type; (+)—more than 50% of wild-type *—Found in cancers: +—less than 10 times; ++—10–30; +++—more than 30. Sources of information: cBioPortal (http://www.cbioportal.org/) and [2,124]. **—Mutation load: med—<1000 per genome, high—1000–5000; ultra-high—more than 5000—methods of determination of mutation rate or frequency and reference: in vivo mutation in Big Blue mice [138]; 6-tioguanine or ouabain-resistant mutants in cultured embryonic fibroblasts, MEFs [138], ^#^—NGS, mutations per megabase [135]; ***(-)—missing catalytic residue, predicted to be exo^−.^n/a—not applicable; nd—not determined.
