# Correction: Pavlov, Y.I., et al. DNA Polymerases at the Eukaryotic Replication Fork Thirty Years After: Connection to Cancer. *Cancers* 2020, *12*, 3489

**DOI:** 10.3390/cancers13050969

**Published:** 2021-02-26

**Authors:** Youri I. Pavlov, Anna S. Zhuk, Elena I. Stepchenkova

**Affiliations:** 1 Eppley Institute for Research in Cancer and Allied Diseases and Buffett Cancer Center, University of Nebraska Medical Center, Omaha, NE 68198, USA; 2 Department of Genetics and Biotechnology, Saint-Petersburg State University, 199034 Saint-Petersburg, Russia; stepchenkova@gmail.com; 3 International Laboratory of Computer Technologies, ITMO University, 197101 Saint-Petersburg, Russia; ania.zhuk@gmail.com; 4 Laboratory of Mutagenesis and Genetic Toxicology, Vavilov Institute of General Genetics, Saint-Petersburg Branch, Russian Academy of Sciences, 199034 Saint-Petersburg, Russia

The authors wish to make the following corrections to this paper [1] in the three last rows of Table 1 occupying pages 1 and 2 where we detected errors in the names and molecular weights of subunits of human pol ζ. The original version of Table 1 is:

The table should be replaced with the following Table 1 with correct information in the three last rows and highlighted in bold the rows with information about catalytic subunits:

**Table 1:** Nomenclature of subunits of yeast and human B-family DNA polymerases *.

Polymerase	Subunit **	Yeast, *S. cerevisiae*			Human, *H. sapiens*		
		Gene	Protein, (Size, kDa)	Also Known As:	Gene	Protein, (Size, kDa)	Also Known As:
primase -pol α	**Small (catalytic primase)**	***PRI1***	**Pri1** **(48)**	**YIR008C**	***PRIM1***	**PRIM1** **(50)**	**p48, p49**
	Large of primase	*PRI2*	Pri2 (62)	YKL045W	*PRIM2*	PRIM2 (59)	*PRIM2A,* p58
	**Catalytic**	***POL1***	**Pol1** **(167)**	***CDC17,*** ***CRT5,*** ***HPR3,*** **YNL102W**	***POLA1***	**POLA1** **(166)**	***POLA, NSX,*** **p180**
	B-subunit	*POL12*	Pol12 (79)	YBL035C	*POLA2*	POLA2 (66)	*FLJ21662,* p70
pol ε	**Catalytic**	***POL2***	**Pol2** **(256)**	***DUN2,*** **YNL262W**	***POLE***	**POLE** **(262)**	***FILS,*** ***POLE1,*** ***IMADEI,*** ***CRCS12,*** **p261**
	B-subunit	*DPB2*	Dpb2 (78)	YPR175W	*POLE2*	POLE2 (60)	*DPE2,* p59
	Third	*DPB3*	Dpb3 (23)	YBR278W	*POLE3*	POLE3 (17)	*CHRAC2,* *YBL1,* p17
	Fourth	*DPB4*	Dpb4 (22)	YDR121W	*POLE4*	POLE4 (12)	*YHHQ1,* p12
pol δ	**Catalytic**	***POL3***	**Pol3** **(125)**	***CDC2, HPR2, TEX1,*** **YDL102W**	***POLD1***	**POLD1** **(124)**	***CDC2, MDPL, CRCS10,*** **p125**
	B-subunit	*POL31*	Pol31 (55)	*HYS2, HUS2, SDP5,* YJR006W	*POLD2*	POLD2 (51)	p50
	Third	*POL32*	Pol32 (40)	*REV5,* YJR043C	*POLD3*	POLD3 (51)	*PPP1R128, KIAA0039,* p66
	Fourth	*-*	*-*		*POLD4*	POLD4 (12)	*POLDS,* p12
pol ζ	**Catalytic**	***REV3***	**Rev3** **(173)**	***PSO1,*** **YPL167C**	***REV3L***	**REV3L** **(353)**	***REV3,*** ***HREV3, POLZ*** **p353**
	Second	*REV7*	Rev7 (29)	YIL139C	*MAD2L2*	MAD2L2 (24)	*hREV7,* p30, *FANCV,* *MAD2B,* *POLZ2*
	B-subunit	*POL31*	Pol31 (55)	*HYS2, HUS2, SDP5*	*POLD2*	POLD2 (51)	p50
	Fourth	*POL32*	Pol32 (40)	*REV5*	*POLD3*	POLD3 (51)	*PPP1R128,* p66

*—mouse gene symbols are the same as humans but written using different capitalization: example for a gene is *Pole*, for a protein—POLe. **—the information on catalytic subunits is highlighted by bold font.

We stress that these errors were purely due to human error and oversight, all corrections done do not change the written portion of the table, or the final conclusion of this manuscript. The manuscript will be updated. The authors would like to apologize for any inconvenience caused. All changes have been reviewed and approved by the Academic Editors.

## Figures and Tables

**Table 1:** Nomenclature of subunits of yeast and human B-family DNA polymerases *.

Polymerase	Subunit **	Yeast, *S. cerevisiae*			Human, *H. sapiens*		
		Gene	Protein, (Size, kDa)	Also Known As:	Gene	Protein, (Size, kDa)	Also Known As:
primase -pol α	Small (catalytic primase)	*PRI1*	Pri1 (48)	YIR008C	*PRIM1*	PRIM1 (50)	p48, p49
	Large of primase	*PRI2*	Pri2 (62)	YKL045W	*PRIM2*	PRIM2 (59)	*PRIM2A,* p58
	Catalytic	*POL1*	Pol1 (167)	*CDC17,* *CRT5,* *HPR3,* YNL102W	*POLA1*	POLA1 (166)	*POLA, NSX,* p180
	B-subunit	*POL12*	Pol12 (79)	YBL035C	*POLA2*	POLA2 (66)	*FLJ21662,* p70
pol ε	Catalytic	*POL2*	Pol2 (256)	*DUN2,* YNL262W	*POLE*	POLE (262)	*FILS,* *POLE1,* *IMADEI,* *CRCS12,* p261
	B-subunit	*DPB2*	Dpb2 (78)	YPR175W	*POLE2*	POLE2 (60)	*DPE2,* p59
	Third	*DPB3*	Dpb3 (23)	YBR278W	*POLE3*	POLE3 (17)	*CHRAC2,* *YBL1,* p17
	Fourth	*DPB4*	Dpb4 (22)	YDR121W	*POLE4*	POLE4 (12)	*YHHQ1,* p12
pol δ	Catalytic	*POL3*	Pol3 (125)	*CDC2,* *HPR2,* *TEX1,* YDL102W	*POLD1*	POLD1 (124)	*CDC2, MDPL,* *CRCS10,* p125
	B-subunit	*POL31*	Pol31 (55)	*HYS2,* *HUS2,* *SDP5,* YJR006W	*POLD2*	POLD2 (51)	p50
	Third	*POL32*	Pol32 (40)	*REV5,* YJR043C	*POLD3*	POLD3 (51)	*PPP1R128, KIAA0039,* p66
	Fourth	*-*	*-*		*POLD4*	POLD4 (12)	*POLDS,* p12
pol ζ	Catalytic	*REV3*	Rev3 (173)	*PSO1,* YPL167C	*REV3L*	REV3L (353)	*REV3,* *HREV3,* *POLZ* p353
	Second	*REV7*	Rev7 (29)	YIL139C	*hREV7*	HREV7 (24)	*MAD2L2,* p30, *REV7,* *FANCV,* *MAD28,* *POLZ2,*
	B-subunit	*POL31*	Pol31 (55)	*HYS2,* *HUS2,* *SDP5*	*POLD3*	POLD3 (50)	p50
	Fourth	*POL32*	Pol32 (40)	*REV5*	*POLD4*	POLD4 (12)	*PPP1R128,* p66

*—mouse gene symbols are the same as humans but written using different capitalization: example for a gene is *Pole*, for a protein—POLe. **—the information on catalytic subunits is highlighted by bold font.
